# Memcapacitor Crossbar Array with Charge Trap NAND Flash Structure for Neuromorphic Computing

**DOI:** 10.1002/advs.202303817

**Published:** 2023-09-26

**Authors:** Sungmin Hwang, Junsu Yu, Min Suk Song, Hwiho Hwang, Hyungjin Kim

**Affiliations:** ^1^ Department of AI Semiconductor Engineering Korea University Sejong 30019 South Korea; ^2^ Department of Electrical and Computer Engineering Seoul National University Seoul 08826 South Korea; ^3^ Department of Electrical and Computer Engineering Inha University Incheon 22212 South Korea

**Keywords:** charge trap flash, crossbar array, memcapacitor, NAND flash structure, neuromorphic computing, spiking neural network

## Abstract

The progress of artificial intelligence and the development of large‐scale neural networks have significantly increased computational costs and energy consumption. To address these challenges, researchers are exploring low‐power neural network implementation approaches and neuromorphic computing systems are being highlighted as potential candidates. Specifically, the development of high‐density and reliable synaptic devices, which are the key elements of neuromorphic systems, is of particular interest. In this study, an 8 × 16 memcapacitor crossbar array that combines the technological maturity of flash cells with the advantages of NAND flash array structure is presented. The analog properties of the array with high reliability are experimentally demonstrated, and vector‐matrix multiplication with extremely low error is successfully performed. Additionally, with the capability of weight fine‐tuning characteristics, a spiking neural network for CIFAR‐10 classification via off‐chip learning at the wafer level is implemented. These experimental results demonstrate a high level of accuracy of 92.11%, with less than a 1.13% difference compared to software‐based neural networks (93.24%).

## Graphical abstract

Memcapacitor crossbar array based on NAND flash array structure is demonstrated for neuromorphic computing. A MOS capacitor is utilized as a memcapacitor with a charge‐trapping layer. Memcapacitive array operations are experimentally verified including vector‐matrix multiplication, and hardware SNN is demonstrated based on VGGNet‐7 for CIFAR‐10 classification.

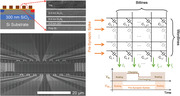

## 1 Introduction

Artificial intelligence (AI), developed rapidly in recent years, fertilizes human society by making a dramatic increase in all kinds of data processing capabilities.^[^ ^1^, ^2^, ^3^, ^4^ ^]^ As a matter of fact, the progress of AI inevitably accompanies the enlargement of the AI model. For instance, OpenAI's GPT‐3—which has made remarkable progress in natural language processing and can even write a basic program code—requires 175 billion parameters, and 1.6 trillion parameters are utilized in the case of Google's Switch Transformer.^[^ ^5^, ^6^ ^]^ Also, this trend is expected to continue as the effectiveness of large‐scale neural networks is proven.^[^ ^7^ ^]^ Therefore, tremendous computational cost and energy consumption are required to train the state‐of‐the‐art model, highlighting the importance of low‐power neural network implementation.^[^ ^8^ ^]^ Consequently, edge‐oriented computing is emerging along with the decentralization of the computing paradigm due to limitations including signal communication traffic induced by von Neumann bottleneck.^[^ ^9^ ^]^ Energy‐efficient neural network implementations are also attracting attention for performing these compute‐intensive tasks in resource‐constrained environments.^[^ ^10^ ^]^ Thus, researchers are focusing on low‐power neural network implementation approaches, and hardware‐based neuromorphic computing systems are highlighted as potential candidates.^[^ ^11^, ^12^, ^13^, ^14^, ^15^, ^16^, ^17^, ^18^ ^]^ A neuromorphic computing system is an architecture that overcomes the limitations of von Neumann computing structures inspired by biological neural systems, where synapses and neurons are massively integrated in parallel.^[^ ^19^, ^20^ ^]^ Synapses store weight information and generate output signals by multiplying inputs with weights, and neurons integrate signals received from synapses and send them as output signals. In this architecture, vector‐matrix multiplication (VMM) can be efficiently and simultaneously conducted by maximized parallel connections. Furthermore, as an event‐driven asynchronous system rather than a clock‐based synchronous system, it is energy‐efficient because the input is spatiotemporally sparse.^[^ ^21^, ^22^ ^]^

In general, memristor devices based on resistance switching behaviors, such as resistive random‐access memory (RRAM), spin‐transfer torque magnetoresistive random access memory (STT‐MRAM), and phase‐change random access memory (PCM), are considered a synaptic component in a neural network.^[^ ^23^, ^24^, ^25^, ^26^, ^27^, ^28^, ^29^ ^]^ Since they are implemented in a crossbar array consisting of two‐terminal devices, they have the advantages of simple fabrication, high cell density, intuitive VMM operation, and easy implementation of a large‐scale array. However, there is a drawback that an error occurs in the current summation due to a problem such as sneak current, which requires additional active selecting components and results in one‐selector‐one‐resistor (1S‐1R) or one‐transistor‐one‐resistor (1T‐1R) array structures.^[^ ^30^, ^31^, ^32^, ^33^, ^34^ ^]^ In contrast, transistor‐type synaptic devices are free from these issues thanks to the gate electrode,^[^ ^35^, ^36^, ^37^, ^38^ ^]^ and these devices are typically integrated in NAND or NOR array structures. NOR‐type structure offers the advantage of parallel matrix operations, similar to artificial neural networks (ANNs), but requires individual drain contacts for each cell, resulting in an area inefficiency of over 10F^2^. Conversely, NAND‐type structures occupy an area of 4F^2^, providing better area efficiency but lack parallel matrix operations due to their serial connection structure.^[^ ^39^ ^]^

Memcapacitive devices based on capacitive coupling have been recently studied due to crossbar array integration by their two‐terminal structure.^[^ ^40^ ^]^ Thanks to their efficient prevention of sneak current through the open‐circuit nature of a capacitor, additional selecting device, such as a transistor or a selector, is not necessary for a memcapacitor array configuration. Furthermore, memcapacitors have the advantage of possessing a significantly high effective resistance, making them less susceptible to performance degradation by line resistance. Also, the high static power inherent in a resistance‐based array system can be effectively eliminated, as capacitors consume only dynamic power. Experimental implementations of memcapacitor crossbar arrays have mainly utilized ferroelectric switching‐based capacitors due to their low switching voltage and fast switching speed.^[^ ^41^ ^]^ However, it has limitations due to a lower multi‐level capability according to scaling down, lower reliability compared to charge trap flash (CTF) memory, and a larger cell density over 4F^2^ to form separate doped silicon regions.^[^ ^42^ ^]^ In some previous studies, simple images were created in‐house to implement pattern recognition applications in fabricated synaptic arrays.^[^ ^42^, ^43^ ^]^ However, it is straightforward to classify such simple patterns, making it challenging to precisely assess the performance of hardware‐based neural networks. Furthermore, the absence of a standard dataset for evaluating neural network performance in these studies makes it difficult to compare their results. Some studies have relied on modeling a single cell rather than fabricating a synapse array to construct the neural network,^[^ ^44^, ^45^ ^]^ which limits the experiments to simulations for the demonstration of cognitive functions.

In this article, we present an 8 × 16 memcapacitor crossbar array based on the NAND flash architecture with a charge‐trapping layer. It aims to combine the high cell density of a crossbar array with the technological maturity of CTF cells for reliable operations. Our proposed device maintains the small footprint of the NAND array (4F^2^) while enabling program (PGM)/erase (ERS) of individual cells and parallel VMM processing, facilitating accurate weight transfer and VMM operations. Additionally, we experimentally demonstrate an energy‐efficient hardware neural network by applying systematic techniques to minimize accuracy loss in converting ANNs to spiking neural networks (SNNs) using the fabricated memcapacitor crossbar array for CIFAR‐10 classification.

## 2 Results and Discussion

### 2.1 Device Fabrication

**Figure 1:**
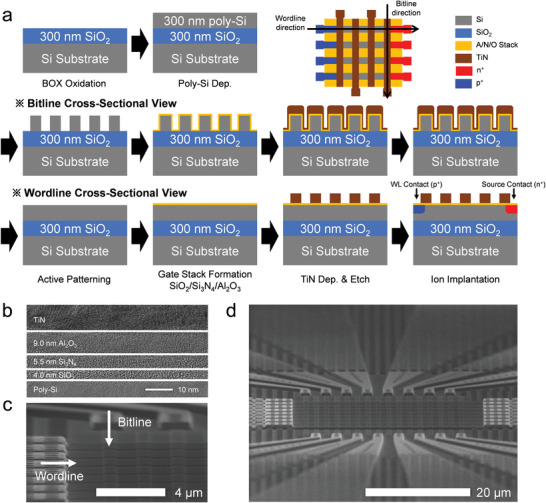
Device fabrication. a) BL and WL cross‐sectional views of the fabrication process. b) Transmission electron microscopy (TEM) image of a gate stack in a memcapacitor. c) Scanning electron microscopy (SEM) image of a fragment of fabricated memcapacitor array. Individual memcapacitors are located at the cross point of BLs and WLs. d) SEM image of 8 × 16 memcapacitor crossbar array.

The fabrication process of the memcapacitor crossbar array is shown in **Figure** **1**a. First, 300 nm thick buried oxide (BOX) was formed on a bulk silicon wafer by wet oxidation using a furnace at 1000 °C for 55 min. Subsequently, a polysilicon substrate of 300 nm thick was deposited by low‐pressure chemical vapor deposition (LPCVD) at 625 °C and 150 mTorr using SiH_4_ of 60 sccm. B^+^ ions for body doping were implanted with a dose of 5 × 10^13^ cm^−2^, followed by drive‐in at 1050 °C for 3 h. After etching the active region, medium temperature oxide (MTO) of 4 nm thick was deposited as a tunneling oxide by LPCVD at 782 °C and 350 mTorr using SiH_2_Cl_2_ of 40 sccm and N_2_O of 160 sccm. Si_3_N_4_ of 5.5 nm was deposited as a charge‐trapping layer by LPCVD at 785 °C and 200 mTorr using dichlorosilane (DCS, SiH_2_Cl_2_) of 30 sccm and NH_3_ of 100 sccm. Then, atomic layer deposition (ALD) was used for 9 nm thick Al_2_O_3_ layer deposition as a blocking oxide using trimethylaluminum (TMA, Al(CH_3_)_3_) and H_2_O as precursors. A TiN metal gate of 50 nm was deposited by sputtering, and 25 nm thick SiO_2_ was deposited as a hard mask by PECVD using tetraethoxysilane (TEOS, Si(OC_2_H5)_4_) of 700 sccm and O_2_ of 700 sccm at 380 °C. After etching the hard mask by dry etch with CHF_3_ of 25 sccm, SF_4_ of 5 sccm, and Ar of 70 sccm and wet etch with buffered oxide etchant (BOE), the TiN gate was etched by wet etch process using H_2_O_2_:DI water (=1:4) solution at 60 °C for 45 min in order to completely remove metal sidewall along with the active area. As^+^ (dose: 5 × 10^15^ cm^−2^, energy: 30 keV) and BF_2_^+^ (dose: 5 × 10^15^ cm^−2^, energy: 30 keV) ions were implanted to form source and substrate contact regions, respectively. Finally, a conventional back‐end‐of‐line (BEOL) process consisting of inter‐layer‐dielectric, contact hole formation, and metallization was carried out. Figure 1b shows a transmission electron microscopy (TEM) image of the fabricated memcapacitor based on a CTF cell positioned at each cross‐point, confirming that TiN/Al_2_O_3_/Si_3_N_4_/SiO_2_/poly‐Si (TANOS) stack was successfully formed. In Figure 1c,d, scanning electron microscopy (SEM) images confirm that bitlines (BLs) and wordlines (WLs) were fabricated in the 8 × 16 crossbar structure, where each cell was a size of 1 × 5 µm^2^. In this context, we designated the substrate electrode receiving the pre‐synaptic signal as WLs and the gate connected to the post‐synaptic neuron as BLs, unlike conventional NAND flash electrode names.

### 2.2 Device Characterization

**Figure 2:**
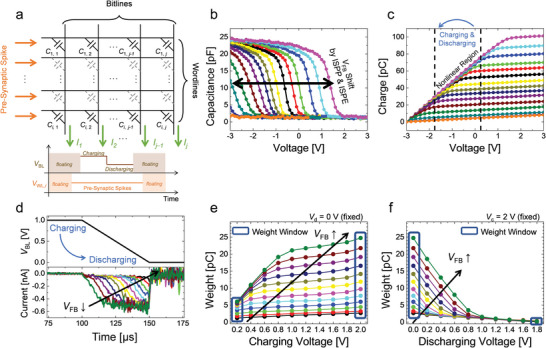
Electrical characteristics of memcapacitor device based on CTF. a) Readout scheme of memcapacitor crossbar array for VMM. Only the charge stored in memcapacitors on selected WL contributes to the parallel summation of BL current during the discharge stage. b) Measured *C*–*V* characteristics with *V*_FB_ shift by ISPP and ISPE methods. c) *Q*–*V* characteristics extracted by integrating *C*–*V* curve depending on the device state. The non‐linear transition region is apparent as the memcapacitor switches from accumulation to depletion state. d) Transient charging and discharging current characteristics according to the memcapacitor state. A change in the current occurred when the memcapacitor was charged and discharged by voltages that covered the non‐linear region. e,f) Extracted weight values by varying *V*_c_ or *V*_d_ to find the optimal read condition for a sufficiently large weight window.

**Figure** **2**a depicts the readout scheme of the memcapacitor crossbar array for VMM computations. The pulse timing diagram shows that pre‐synaptic spikes, *V*_in,i_, are applied to *i*‐th WL, while the unselected WLs are in a floating state. During this time period, the BL voltage (*V*_BL_) is charged to the charging voltage (*V*_c_), and charge accumulation occurs only across the memcapacitors in the selected WLs because the floating state ensures that the unselected memcapacitors remain uncharged. Afterward, as the BL discharges from *V*_c_ to the discharging voltage (*V*_d_), charge *Q*_j_ of the *j*‐th BL flows out of BL as a form of current *I_j_* (*t*), which may be expressed using the following Equation (1):

(1) $$\begin{array}{ccc} Q_{j} & = & \sum_{i}\delta_{i}\int C_{i,j}dV=\;\int I_{j}\left(t\right)dt\;\mathrm{where}\;\delta_{i}\\ & = & \left\{\begin{array}{c} 0,\;if\;V_{\mathrm{WL},\;i}\;is\;floating\;\\ 1,\;otherwise\; \end{array}\right. \end{array}$$

Equation (1) shows that the VMM operation can be conducted by *Q*_j_ when *δ*_i_ and $\int C_{i,j}dV$ represent the pre‐synaptic spikes and synaptic weight, respectively. A synaptic weight is defined as $\int C_{i,j}dV$ instead of *C*_i,j_ since it involves the nonlinear capacitance‐voltage (*C*–*V*) characteristics of a MOS capacitor. All BLs can be read simultaneously, making parallel computation possible for artificial neural networks. The electrical characteristics of the memcapacitor cell were verified to validate the operation scheme of the memcapacitor crossbar array. Firstly, the *C*–*V* characteristics were measured depending on the device state at a high frequency of 100 kHZ, as shown in Figure 2b, confirming that the flat‐band voltage (*V*_FB_) can be shifted by the incremental step pulse program (ISPP) and erase (ISPE) schemes. Figure 2c illustrates the charge‐voltage (*Q*–*V*) characteristics obtained by integrating the *C*–*V* curves. Due to capacitance changes occurring when the surface state of the substrate transitions from accumulation (*C*_ox_) to depletion (*C*_dep_) states in the *C*–*V* characteristics, a nonlinear region shifts in response to PGM and ERS states. By selecting proper *V*_c_ and *V*_d_ to cover the depletion region, the synaptic weight can be extracted as ***w*** = *Q*(*V*_c_)–*Q*(*V*_d_) (see Figure S1, Supporting Information, for more details).

The readout operation was demonstrated by measuring the discharging current (*I*_dis_) according to PGM states, as shown in Figure 2d. When *V*_c_ was applied to the WL electrode, it induced a charge *Q*(*V*_c_) across the capacitor. Subsequently, when the WL voltage was reduced to *V*_d_, the capacitor discharged to the charge *Q*(*V*_d_), causing the *I*_dis_ to flow out of the BL. It is confirmed that *I*_dis_ was decreased with respect to the decrease in *V*_FB_ since the capacitance value at *V*_d_ becomes smaller (depletion region), and the synaptic weight was obtained by integrating the *I*_dis_ with respect to time. Since the weight window (***w***_max_–***w***_min_) is determined by the read condition (*V*_c_ and *V*_d_) during the readout operation, the weight values were measured and extracted under different device states by varying either *V*_d_ or *V*_c_, as shown in Figure 2e,f. The characteristics of weight window, weight fine‐tuning, and energy consumption depend on *V*_c_ and *V*_d_. A large voltage difference (*V*_c_–*V*_d_) is required to achieve a larger weight window (***w***_max_–***w***_min_) by utilizing the wide region of *C*–*V* curves from depletion to accumulation with the same amount of *V*_FB_ shift. It allows accurate weight fine‐tuning, but it can increase energy consumption due to the high operation voltage. Conversely, a small *V*_c_–*V*_d_ can reduce the energy consumption, but a more precise fine‐tuning process is required for weight modulation due to a narrow *V*_FB_ shift region, which can increase the number of tuning cycles and lead to an increase in the electrical stress of the memcapacitor. As depicted in Figure 2e, the increase in the weight window reaches a saturation point with increasing *V*_c_ while *V*_d_ is fixed at 0 V because a higher *V*_c_ extends to the minimum capacitance region. Hence, raising *V*_c_ too high is unnecessary, and memory windows exceeding a certain threshold become redundant. To achieve a wider weight window while maintaining a low operating voltage, *V*_C_ and *V*_D_ were set to 1 and 0 V, respectively, during the readout operations.

Based on the operation principle of the proposed memcapacitor, it is necessary to have a significant difference between the capacitances of the depletion (*C*_dep_) and accumulation (*C*_ox_) states to obtain stable and precise multi‐bit operation with a higher weight window. From a device fabrication perspective, achieving a large *C*_ox_ requires a thin gate stack with a high dielectric constant, while a low *C*_dep_ demands a thick active layer and low body doping concentration. However, gate stack engineering should be carefully carried out for the device memory functions as a CTF cell. Also, if the doping concentration of the active region is too low, accessing a specific cell becomes difficult due to the high resistance of the selected active region when the target cell is fully depleted. This resistance issue becomes more pronounced when multiple cells are selected on the same BL. Therefore, it is required to properly determine the thickness and doping concentration of the active layer, considering the trade‐off relationship between these resistance issues and memory characteristics. With optimization in these aspects, it is expected that nanometer‐scale device scaling down could be achieved with a proportional decrease in energy consumption.

### 2.3 Array Operation

**Figure 3:**
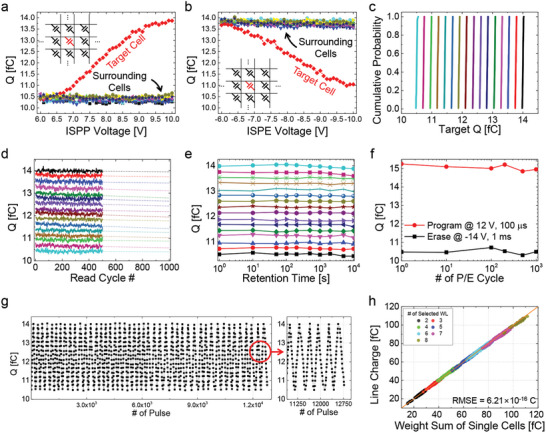
Switching and VMM characteristics of 8 × 16 memcapacitor crossbar array. a,b) Weight modulations of target cells and surrounding cells. The target cells were programmed and erased using ISPP and ISPE, while the surrounding cells were inhibited. c) Weight fine‐tuning results for all devices in the memcapacitor array, with equally spaced 4‐bit levels between 10.5 and 14 fC. d) Read disturbance results for 4‐bit levels measured for up to 500 cycles and extrapolated up to 10^3^ cycles. e) Retention characteristics of 4‐bit weight levels measured at room temperature (300 K) for 10^4^ s. f) Cycling endurance characteristics measured by repeatedly applying *V*_PGM_ of 12 V and 100 µs and *V*_ERS_ of −14 V and 1 ms, switching the device over 10^3^ cycles between its two end‐states. g) Cycling endurance characteristics of the multi‐level readout, measured by gradually increasing and decreasing the 4‐bit levels through weight fine‐tuning. Even after more than 10^4^ repetitive pulses, the weight could be fine‐tuned properly. h) VMM results when different numbers of WLs were randomly selected. The sum of the weights read through individual access (on the *x*‐axis) and the BL charges when those cells were read simultaneously (on the *y*‐axis) showed a substantially accurate linear relationship.

To characterize the electrical properties of the fabricated 8 × 16 memcapacitor array, we used different PGM and ERS schemes compared to conventional NAND flash memory, as each WL has an independent substrate contact (see “Experimental Setup” section).^[^ ^46^ ^]^ It is important that surrounding cells maintain their states during the PGM or ERS operation of a selected cell to ensure the accurate transfer of pre‐trained weights onto the fabricated array. To verify the effectiveness of the PGM/ERS schemes including disturbance, the changes in the weight value of a selected target cell and surrounding unselected 8 cells in 3 × 3 subarray were measured during ISPP and ISPE operations. The read condition used for extracting synaptic weights was *V*_c_ of 1.0 V and *V*_d_ of 0.0 V. Starting from the fully erased state of all the memcapacitors, only the target cell was programmed by ISPP program voltage (*V*_PGM_) from 6.0 to 10.0 V in steps of 0.1 V. **Figure** **3**a confirms that the states of the surrounding cells kept the same while only the target cell weight was increased. Similarly, the target cell was erased by ISPE erase voltage (*V*_ERS_) from −6.0 to −10.0 V in steps of −0.1 V, with all cells initially fully programmed. It was observed that only the target cell weight was decreased whereas there was no change in the synaptic weights of the surrounding cells, as shown in Figure 3b. These results verify that the proposed PGM/ERS schemes are effective in selectively modifying the synaptic weights of target cells without affecting the surrounding cells.

For reliable and accurate inference operations in a neuromorphic system, it is necessary to accurately set the device weight to the desired target value, and we employed a closed‐loop weight fine‐tuning scheme (see Figure S2, Supporting Information). Starting with an initial PGM/ERS voltage of ±6 V, a pulse step of ±0.1 V, and cycle number *n* = 0, the current weight state *Q* was verified under the read condition *V*_c_ = 1.0 V and *V*_d_ = 0.0 V. The weight fine‐tuning was completed when the criterion (*Q*_t_–Δ*Q* < *Q* < *Q*_t_ + Δ*Q*) was met, where *Q*_t_ and Δ*Q* are the target weight and an error margin, respectively. Otherwise, *V*_PGM_ or *V*_ERS_ was applied to change the weight state depending on the current state. The fine‐tuning was considered a failure if the maximum cycle (*N* = 200) or maximum PGM/ERS voltage (±10.0 V) was exceeded, which was determined considering the device reliability. To get linearly separated 4‐bit level weight values in the memcapacitor, the weight fine‐tuning operation was performed with the error margin (Δ*Q*) of 0.025 fC for a whole memcapacitor crossbar array. As a result, the cumulative probability distribution in Figure 3c confirms that all the cells were accurately adjusted to the target 4‐bit state. On average, 23 fine‐tuning pulses were required to adjust the synaptic weight from the previous state to the next state (see Figure S3, Supporting Information).

In addition, the reliability characteristics including endurance and retention were investigated since the performance of neural networks can be significantly affected. Above all, the read disturbance of the 4‐bit level weight state for 500 read cycles was verified, as shown in Figure 3d. It is observed that there was almost no read disturbance in the fabricated crossbar array under the read condition in the results of extrapolation up to 10^3^ read cycles from the measurement data. Moreover, the retention characteristics of the 4‐bit level weight were experimentally demonstrated at room temperature (300 K), as shown in Figure 3e, confirming that the 4‐bit weight level was still accurately distinguished up to 10^4^ s. We also evaluated the cycling endurance using *V*_PGM_ (12 V, 100 µs) and *V*_ERS_ (−14 V, 1 ms), as depicted in Figure 3f. The weight window remained almost unchanged even after 10^3^ cycles of PGM/ERS operations. Subsequently, the multi‐level cycling endurance for the 4‐bit weight level was evaluated, as illustrated in Figure 3g. The 4‐bit level weight could be constantly manipulated by applying PGM/ERS pulses over 10^4^ times yet exhibited good endurance characteristics.

Finally, to verify the accuracy of VMM operations in various weight mapping scenarios, we randomly selected memcapacitor cells in the crossbar array and programmed some of them with *V*_PGM_ values ranging from +6.0 to +10.0 V, while the others were erased with *V*_ERS_ values ranging from −6.0 to −10.0 V, resulting in a distribution of randomized weights across the array. Pre‐synaptic inputs were then applied to randomly selected WLs simultaneously, emulating the sparsity of SNNs. The charges at BLs, corresponding to the weighted sum, were extracted and compared with the sum of each individual cell weight, as shown in Figure 3h. The correlation diagram shows the weighted sum of individual cells on the *x*‐axis and the BL weighted sum when multiple WLs were selected simultaneously on the *y*‐axis. It shows an excellent correlation between the *x*‐axis and *y*‐axis, with a substantially small root‐mean‐squared error (RMSE) of 0.62 fC, meaning that the VMM operation can be accurately performed in the fabricated memcapacitor array. The correlation was consistent regardless of the number of selected WLs, which indicates that the capacitor coupling impact on the floating node hardly occurred and there was no sneak current issue, which is a common problem in memristor crossbar arrays.

### 2.4 Hardware SNN Demo

**Figure 4:**
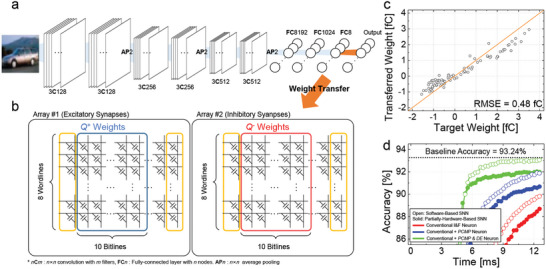
Implementation of a hardware‐based SNN for CIFAR‐10 classification. a) Illustration of a modified VGGNet‐7. b) Schematic diagram of a hardware‐based SNN implementation in memcapacitor crossbar arrays. Pre‐trained weights were normalized to accommodate the memcapacitor weight range and transferred to two arrays. c) Correlation diagram between the ideal weights trained by software‐based ANN and the transferred weights in the array. d) Accuracy comparison of software and hardware‐based SNNs according to timestep.

A partially hardware‐based SNN for CIFAR‐10 classification was experimentally demonstrated using the fabricated memcapacitor crossbar array with a slightly modified VGGNet‐7 (see Experimental Section). Specifically, a hidden layer was added with eight neurons right before the output layer, as shown in **Figure** **4**a. While the convolution layers and the early fully connected layers were preprocessed by software, the final 8 × 10 fully connected layer was experimentally implemented in wafer‐level hardware. The performance of the SNN was verified by post‐processing the measured output from the memcapacitor array, assuming that ideal integrate‐and‐fire (I&F) neurons were utilized.^[^ ^47^ ^]^ Two synaptic devices were paired to implement negative weights. Positive weights were implemented by adjusting the excitatory synaptic devices together with inhibitory synaptic devices fixed at the minimum weight. In contrast, negative weights were implemented by adjusting the inhibitory synaptic devices together with excitatory synaptic weights fixed at the minimum weight. Therefore, the weights trained through software were normalized to fit within the memcapacitor weight range (≈ −5 fC < ***w*** < +5 fC) and transferred to two 8 × 10 arrays, as shown in Figure 4b. The weight transfer was performed one by one, resulting in the adjustment of a total of 160 cells in both the excitatory and inhibitory arrays to their target values. When implementing positive weights, the cells in the inhibitory array were fully erased, while for negative weight implementation, the cells in the excitatory array were fully erased. The target weights were then adjusted accordingly. Following this, the 160 cells in the two 8 × 10 arrays were accessed one by one to precisely adjust the weights to their target values. The weighted sum was then experimentally measured by applying pre‐synaptic inputs to the 8‐WL and post‐processing the outputs to determine their accuracy. The I&F neurons were assumed to have a membrane capacitance (*C*_mem_) of 4.7 fF and a threshold of 1.0 V.

After a total of 160 weights, including both excitatory and inhibitory synapses, were transferred to the fabricated memcapacitor array through the weight fine‐tuning procedure, we compared them to the desired target weights, as shown in Figure 4c. The discrepancy between them can come from several factors. During the weight fine‐tuning process, soft PGM/ERS can cause disturbances in cells that have already been fine‐tuned. Despite PGM/ERS inhibition characteristics with respect to ISPP/ISPE, disturbances can occur when applying a large number of pulses required to transfer all 80 synaptic weights on the excitatory and inhibitory arrays. Additionally, the verification process during fine‐tuning involved *C*–*V* measurement, which applies ac small signals to dc bias and can cause disturbances due to the stress caused by the DC bias. Despite these disturbances, it presents a good correlation between the ideal weights and the transferred weights in the memcapacitor array with an RMSE of 0.48 fC. In classification tasks, the decision‐making process only involves the most frequently firing neurons, so it is sufficient to precisely determine the output as long as the correlation is maintained to some extent.

By post‐processing the measured transient discharging current characteristics of all the BLs, we compared the performance of the hardware‐based SNN with that of the ideal software‐based SNN, as shown in Figure 4d. During the inference time of 12 ms, the hardware‐based SNN for image classification achieved an 88.76% accuracy with the conventional I&F neuron model, which is nearly identical to the 89.95% accuracy obtained by software‐based SNN simulation with the ideal target weight. However, there was a large difference compared to the ANN baseline accuracy of 93.24%. This was due to the considerable latency, which prevented the performance from reaching a steady state within 12 ms. To improve the SNN accuracy to that of ANN, we applied pre‐charged membrane potential (*PCMP*) and delayed evaluation (*DE*) techniques to the conventional I&F neuron model, which have been reported to help reduce the inherent latency of SNNs.^[^ ^48^, ^49^ ^]^

When the membrane potential of the I&F neuron was pre‐charged to 0.4 V prior to inference by *PCMP*, a significant reduction in the latency of the hardware‐based SNN was observed, which showed a classification accuracy of 90.71%. This result indicates an accuracy drop of only 1.26%p compared to that of the software‐based SNN (91.97%) with the ideal weights using the same pre‐charging level. We assessed the performance by additionally applying *DE* of ≈4 ms to the hardware‐based SNN. As a result, the accuracy was 92.11% in the hardware demonstration, indicating an additional accuracy improvement of ≈1.40%p (92.11%–90.71% = 1.40%p). Even though the accuracy was reduced by ≈1.08%p compared to that of the software‐based SNN with the ideal weights (93.19%) when the same techniques were used, this is a noteworthy outcome given that wafer‐level hardware demonstration achieved significantly high accuracy close to the ANN baseline accuracy. Consequently, it was demonstrated that the weight transfer was successfully performed, resulting in  an average accuracy drop within 1.18%p relative to the software‐based SNN in all cases.

**Figure 5:**
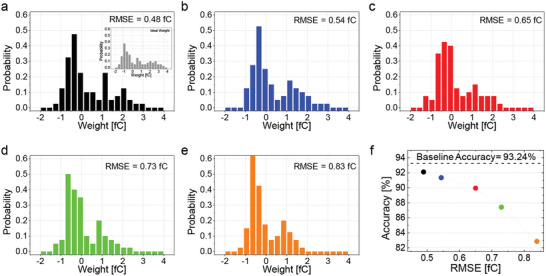
Evaluation of classification accuracy at a steady state with respect to different RMSE values of transferred weight distributions in the fabricated synapse array. a–e) Weight distributions with different RMSE values, ranging from 0.48 to 0.83 fC. The inset in panel **a** shows the ideal pre‐trained weight distribution. f) Evaluation of SNN inference accuracy using weight distributions with different RMSE values and low‐latency SNN techniques, including *PCMP* and *DE*. The highest accuracy was achieved at an RMSE value of 0.48, and the accuracy drop increased with increasing RMSE values.

In addition, we investigated the impact of weight transfer errors on the performance of the hardware‐based SNN. **Figure** **5**a–e presents the weight distributions transferred to the memcapacitor crossbar array with varying RMSE values from 0.48 to 0.83 fC. While all the weight distributions were relatively close to the ideal weight distribution shown in the inset of Figure 5a, the accuracy drop increased as the RMSE value of the transferred weight increased, as illustrated in Figure 5f displaying the best accuracy achieved during SNN inference time of 12 ms with all the techniques for low‐latency SNN (such as *PCMP* and *DE*) applied. It confirms that an accurate weight transfer, which can be obtained by the fine‐tuning process and charge‐trapping layer, is required for the high performance of hardware‐based neural networks. Additionally, if the entire network were implemented in hardware instead of a subset with the same level of error, a greater decrease in accuracy would be expected; therefore, a highly accurate weight control capability is essential to achieve high‐performance hardware‐based SNNs (see Figure S4, Supporting Information).

## 3 Conclusion

In this paper, we have demonstrated the feasibility of the memcapacitor crossbar array based on CTF and its potential for implementing hardware SNNs. Despite its structural similarity to NAND flash and high integration density of 4F^2^, which was fabricated using standard Si CMOS processes, this array enabled simultaneous parallel VMM computations by operating based on capacitive coupling behaviors rather than transistor‐based operations. The weight modulation of the memcapacitor devices within the crossbar array and the readout scheme based on charging and discharging were verified. Moreover, we confirmed the PGM/ERS operation characteristics of the whole crossbar array, including inhibition and disturbance, and demonstrated accurate VMM operations within the memcapacitor crossbar array. Finally, we implemented the hardware SNN in the memcapacitor crossbar array and achieved nearly the same CIFAR‐10 recognition performance as the baseline accuracy, thanks to the accurate weight adjustment and reliability characteristics of CTF cells. The experimental validation of the memcapacitor crossbar array with 4F^2^ cell density using CTF is expected to lead to stable and accurate neuromorphic computing systems.

## 4 Experimental Section

### Measurement Details

To characterize the memcapacitor devices, electrical measurements were conducted using various modules of the Keysight B1500A parameter analyzer, including the source measure unit (SMU), high voltage semiconductor pulse generator unit (HV‐SPGU), multi‐frequency capacitance measurement unit (MFCMU), and waveform generator/fast measurement unit (WGFMU). Especially, ISPP/ISPE was applied to the memcapacitor to confirm *V*_FB_ shift through *C‐* *V* measurements. Then, the synaptic weight was extracted by integrating the current over time, which was obtained from transient measurement using the WGFMU. For the characterizations of a whole array, the Keysight E5250A low‐leakage switch mainframe and a 32‐channel probe card were used for BL and WL selections. The built‐in programming tool was employed and customized to control both the parameter analyzer and switching matrix.

### Program and Erase Scheme for Memcapacitor Crossbar Array

The fabricated memcapacitor crossbar array had a structural similarity to a NAND flash array, but there were differences in the substrate commonality and dopant types of the source/drain, leading to notable discrepancies during PGM and ERS operations. When comparing PGM operations between a NAND flash array and the fabricated memcapacitor array, differences could be observed in the applied voltage schemes. In a NAND flash array, *V*_PGM_ was applied to the selected word line (WL), and pass voltage (*V*_pass_) was applied to the unselected WL with a grounded substrate. Then, the target cell was programmed while the unselected cells on the same WL were inhibited from programming through self‐boosting. When comparing ERS operations, it is important to note that in a NAND array, all BLs are connected to a common substrate, which makes it impossible to erase individual cells. Therefore, block ERS was performed, which could be disadvantageous for weight transfer because fine‐tuning of weights requires the erasure of individual cells.

In contrast, in the fabricated memcapacitor array, for the BL with the target cell, a sufficiently large positive bias (*V*_PGM_) was applied to trap electrons by FN tunneling, while a sufficiently large negative bias (*V*_ERS_) was applied for hole injection. Simultaneously, the BLs of unselected cells received either +*V*_pass_ or −*V*_pass_, with a magnitude sufficient to induce the channel under the target cell by pulling carriers from the n^+^ or p^+^ region of the substrate, all while avoiding FN tunneling in the unselected cells. Additionally, either the n+ or p+ region was set to 0 V for electron trapping and hole trapping, respectively, while the other was left in a floating state. Conversely, it was essential to inhibit all memcapacitors for BLs without the target cell. In this scenario, preventing channel formation was crucial because certain memcapacitors still experienced the carrier injection voltage (*V*_PGM_ or *V*_ERS_) applied to these BLs. This could be achieved by applying +*V*_pass_ or −*V*_pass_ to the n^+^ or p^+^ region supplying the target carriers, while leaving the other region floating. In this experiment, *V*_PGM_ was employed within a voltage ranging from +6.0 to +10.0 V and *V*_pass_ at 5 V in the PGM state. For the ERS state, *V*_ERS_ was used within a voltage ranging from −6.0 to −10.0 V, with *V*_pass_ set at −3 V. The illustration of the voltage scheme in the memcapacitor array is depicted in Figure S5 (Supporting Information).

### Training ANN for Off‐Chip Learning and SNN Conversion

The trained neural network was a modified VGGNet‐7 composed of 3**C**128‐3**C**128‐**AP**2‐3**C**256‐3**C**256‐**AP**2‐3**C**512‐3**C**512‐**AP**2‐**FC**1024‐**FC**8‐**FC**10 where *n*C*m*, AP*n*, and FC*n* represent *m* convolution filters of size *n* × *n*, average pooling layer of size *n* × *n*, and fully connected layer with *n* neurons, respectively (see Figure 4a). The network was trained on the CIFAR‐10 dataset using stochastic gradient descent with a momentum of 0.9 as the optimizer. The initial learning rate was 0.1, which was decreased by a factor of 0.1 after 120, 220, and 280 epochs. To improve generalization performance and training speed, batch normalization was applied to all layers except for the output layer, and data augmentation was used based on a random 32 × 32 crop from an image padded by four pixels on each side and with horizontal flipping. After training, the network achieved a classification accuracy of 93.24% on the test dataset. The trained weights were normalized using the ANNs‐to‐SNNs conversion method and then rescaled to fit the weight range of the memcapacitor device, making them suitable for the off‐chip training of SNNs.

## Conflict of Interest

The authors declare no conflict of interest.

## Supporting information

Supporting Information

## Data Availability

The data that support the findings of this study are available from the corresponding author upon reasonable request.
