# Supplementary material for: Memcapacitor Crossbar Array with Charge Trap NAND Flash Structure for Neuromorphic Computing
Source: Adv Sci (Weinh). 2023 Sep 26;10(32):2303817. doi: 10.1002/advs.202303817 (PMC10646263; doi:10.1002/advs.202303817)

ADVANCED SCIENCE
Open Access

Supporting Information

for *Adv. Sci.*, DOI 10.1002/advs.202303817

Memcapacitor Crossbar Array with Charge Trap NAND Flash Structure for Neuromorphic Computing

*Sungmin Hwang, Junsu Yu, Min Suk Song, Hwiho Hwang and Hyungjin Kim**

Supporting Information

Title Memcapacitor Crossbar Array with Charge Trap NAND Flash Structure for Neuromorphic Computing

Sungmin Hwang, Junsu Yu, Min Suk Song, Hwiho Hwang, and Hyungjin Kim*

Figure S1. Working principle of memcapacitor with flash cell structure. a *C-V* characteristic of the memcapacitor with a charge trapping layer. The flat band voltage (V_{FB}) shifts depending on the trapped charge. **b** *Q-V* curve of the memcapacitor obtained by integrating *C-V* curve based on its state. Induced charges corresponding to V_c and V_d are depicted. **c** Output charge (Q_{out}) based on the state of the memcapacitor and the charging/discharging phase. For unselected cells, due to the substrate being in a floating state, Q_{out} is always induced to be nearly zero, regardless of the state.

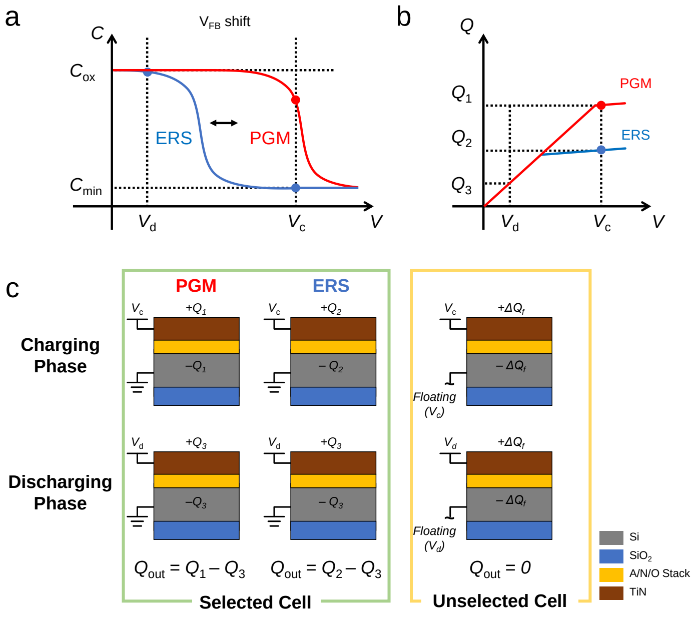

Fundamentally, it utilizes the varying capacitance of a MOS capacitor based on its accumulation and depletion states. In the accumulation state, the maximum capacitance ($C_{max} \approx C_{ox}$) is governed by the oxide capacitance by the A/N/O stack. Meanwhile, the minimum capacitance (C_{min}) occurs when the poly-Si substrate is depleted and is determined by the parallel combination of the oxide capacitance (C_{ox}) and the minimum depletion capacitance (C_{dep}). Additionally, the presence of trapped charges in the memcapacitor cell causes a shift in the flat-band voltage (V_{FB}) in the *C-V* characteristic, as shown in Figure S1a. Since the charging voltage (V_c) and the discharging voltage (V_d) are constants, it leads to a corresponding change in the induced charge on the memcapacitor, which can be represented as the *Q-V* curve, as depicted in Figure S1b. Therefore, when we charge and discharge the memcapacitor, the amount of charge flowing out of the capacitor (Q_{out}) varies depending on the state of the cell. For example, as shown in Figure S1c, in the PGM state, we can obtain $Q_{out} = Q_1 - Q_3$, which corresponds to the synaptic weight, while Q_{out} becomes $Q_2 - Q_3$ in the ERS state. On the other hand, when there is no pre-synaptic spike, the substrate of unselected devices become a floating state, causing negligible charge (ΔQ_f) accumulation on the memcapacitor, resulting in Q_{out} being close to 0, as illustrated in Figure S1c. However, when there is no pre-synaptic spike, the substrate of unselected devices enters a floating state, resulting in negligible charge accumulation (ΔQ_f) on the memcapacitor and Q_{out} being close to 0.

Figure S2. Weight fine-tuning procedure. To start fine-tuning a memcapacitor cell, all conditions are initialized, setting the fine-tuning cycle to 0 and V_{PGM}/V_{ERS} to ±6 V. The current state is then checked using the read condition (V_c of 1V and V_d of 0 V). If the fine-tuning cycle exceeds the set value of 200 or V_{PGM}/V_{ERS} exceeds the limit of ±11 V, the fine-tuning process is considered a failure and the next cell is processed. Otherwise, if the current state is within the acceptable range of error from the target weight, the fine-tuning process is considered a success and the next cell is processed. If not, a 0.1 V step of ISPP/ISPE is applied to change the device state. The last applied V_{PGM} and V_{ERS} are continuously stored during fine-tuning, allowing for quick adjustment from the initial voltage to the target weight in case of a reversed weight change.

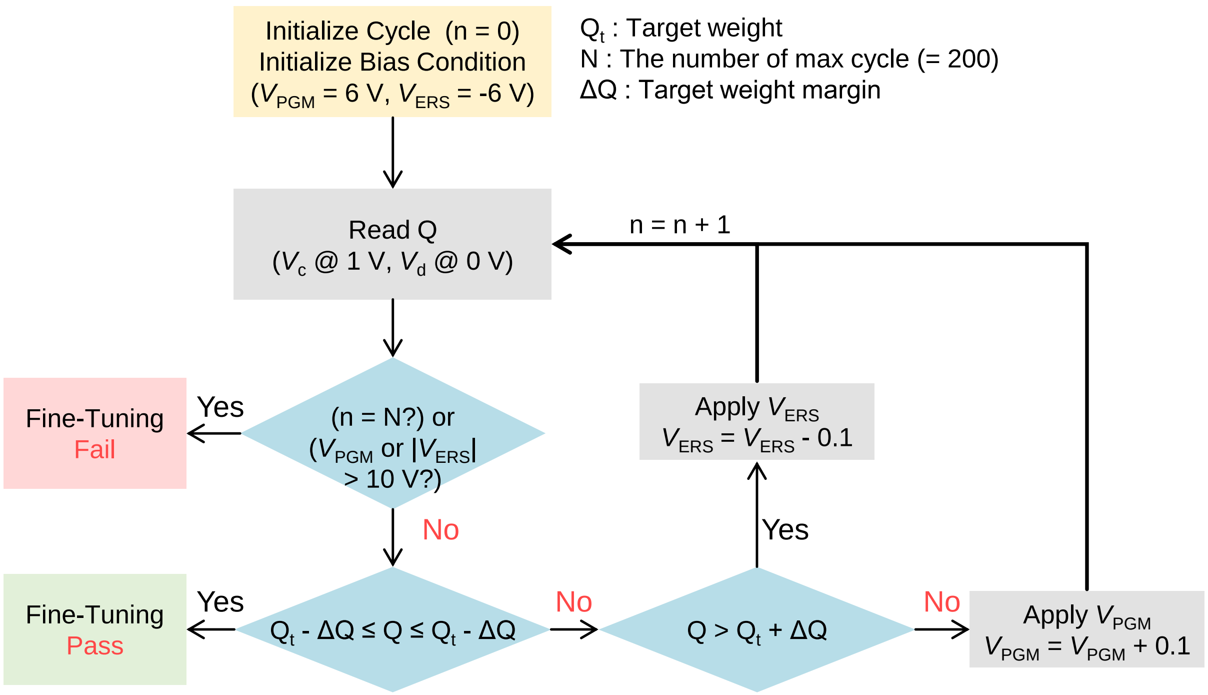

Figure S3. Required number of pulses for weight fine-tuning. During the fine-tuning process of all the devices in the memcapacitor array for 4-bit states (see Figure 3c), we extracted statistics on the total pulse count needed to fine-tune each to the next weight state. As the previous V_{PGM} and V_{ERS} were saved and reused when the direction of weight fine-tuning changed, the target weight was quickly fine-tuned, taking an average of 23 fine-tuning cycles. Note that the number of cycles required to fine-tune a target weight may vary depending on factors such as the weight error margin and the voltage step of ISPP/ISPE.

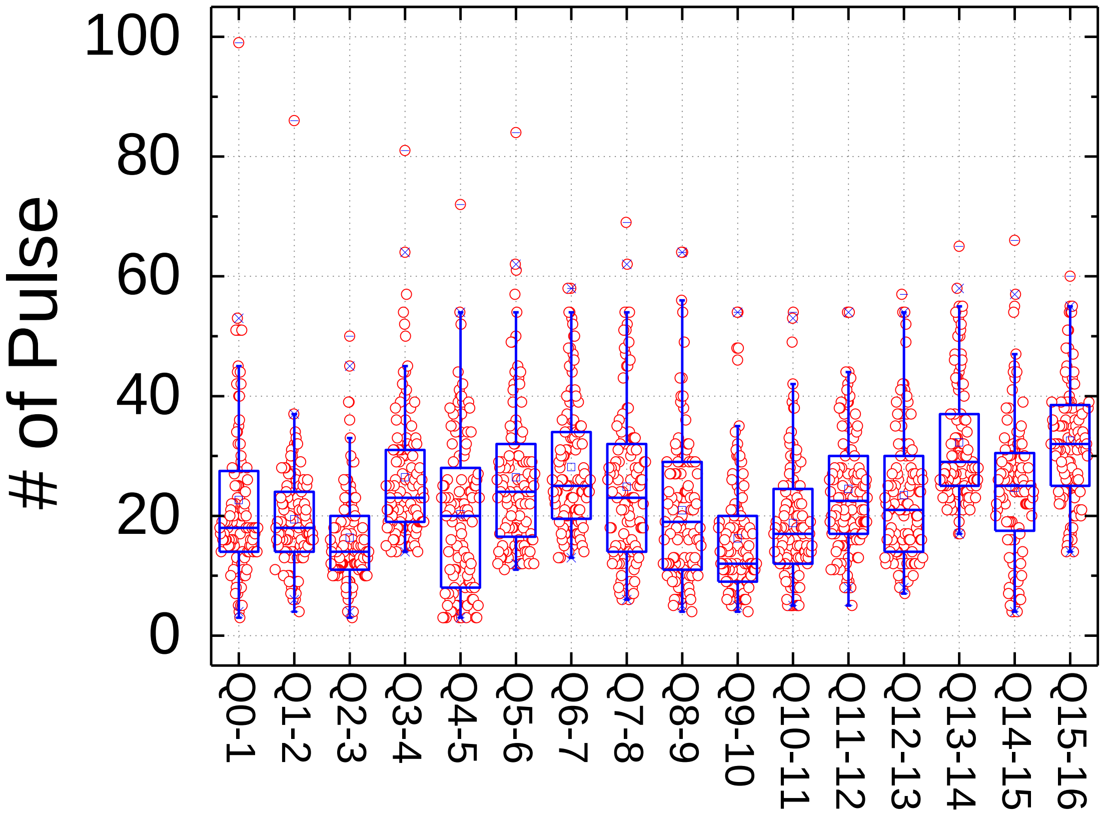

Figure S4. **System-level simulations of VGGNet-7 with weight transfer error. a** System-level simulation results of the modified VGGNet-7 by accumulatively applying the weight transfer error of 0.48 fC to layers from the output to input. **b** Expected classification accuracy when applying weight transfer error reduced by ×2, ×5, ×10, and ×20 times to all layers compared to the reference with an RMSE of 0.48 fC.

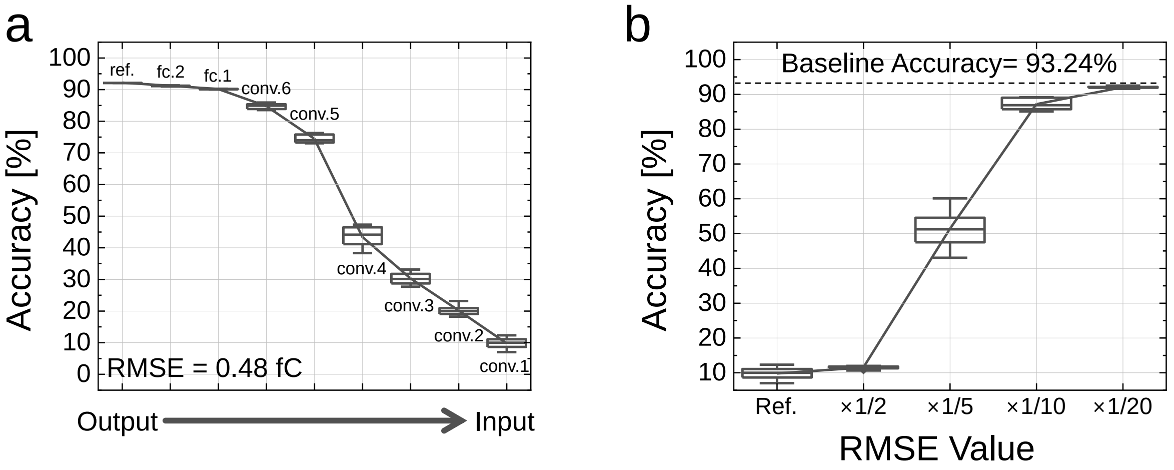

The performance of the hardware SNN was estimated when the measured weight transfer error of 0.48 fC is applied to all other layers. Starting from the output and moving towards the input layer, we evaluated classification accuracy while incrementally applying RMSE values on a layer-by-layer basis. Finally, we assessed the performance by introducing errors of the same magnitude as those in the hardware-transferred weights to all layers. As shown in Figure 4a, when transfer errors were applied only up to the fully-connected layers ('fc.' in Figure 4a), accuracy remained consistently high, exceeding 90%. However, when weight errors started affecting convolutional layers ('conv.' in Figure 4a), a noticeable drop in accuracy occurred. Eventually, it became clear that applying the transfer error to all layers caused the network to malfunction. This is due to the propagation of errors from earlier to later layers as the network becomes deeper. For high-performance hardware-based SNNs, precise weight transfer control is essential. To determine the required reduction in weight transfer error, we conducted additional system-level simulations as shown in Figure 4b. We demonstrated how accuracy changed as RMSE decreased by factors of ×2, ×5, ×10, and ×20 compared to the reference value of 0.48 fC when transfer errors were applied to all layers. The results showed that reducing RMSE by approximately ×5 resulted in a sharp increase in accuracy to around 50%. Accuracy was restored to about 90% with a reduction of ×10 to ×20 in RMSE. In conclusion, the current level of error applied to shallow layers maintained reasonably high accuracy, but accuracy significantly dropped in deeper layers. To address this, a reduction of approximately ×10 to ×20 times in weight transfer error is needed. Achieving this requires smaller voltage steps during the ISPP (or ISPE) scheme for weight fine-tuning, as well as the development of schemes to effectively mitigate soft program and erase phenomena occurring in other cells during fine-tuning.

Figure S5. Program and erase schemes for memcapacitor crossbar array. Schematic diagrams illustrate the different bias conditions of memcapacitor array, including **a** program, **b** program inhibit, **c** erase, and **d** erase inhibit states. A program method similar to a conventional NAND flash array can be employed thanks to n^+ doped region. In contrast, unlike a NAND flash memory, it allows for the individual erasure of cells thanks to p^+ doped region instead of block erase, enabling the fine-tuning of each cell's weight without affecting other cells.

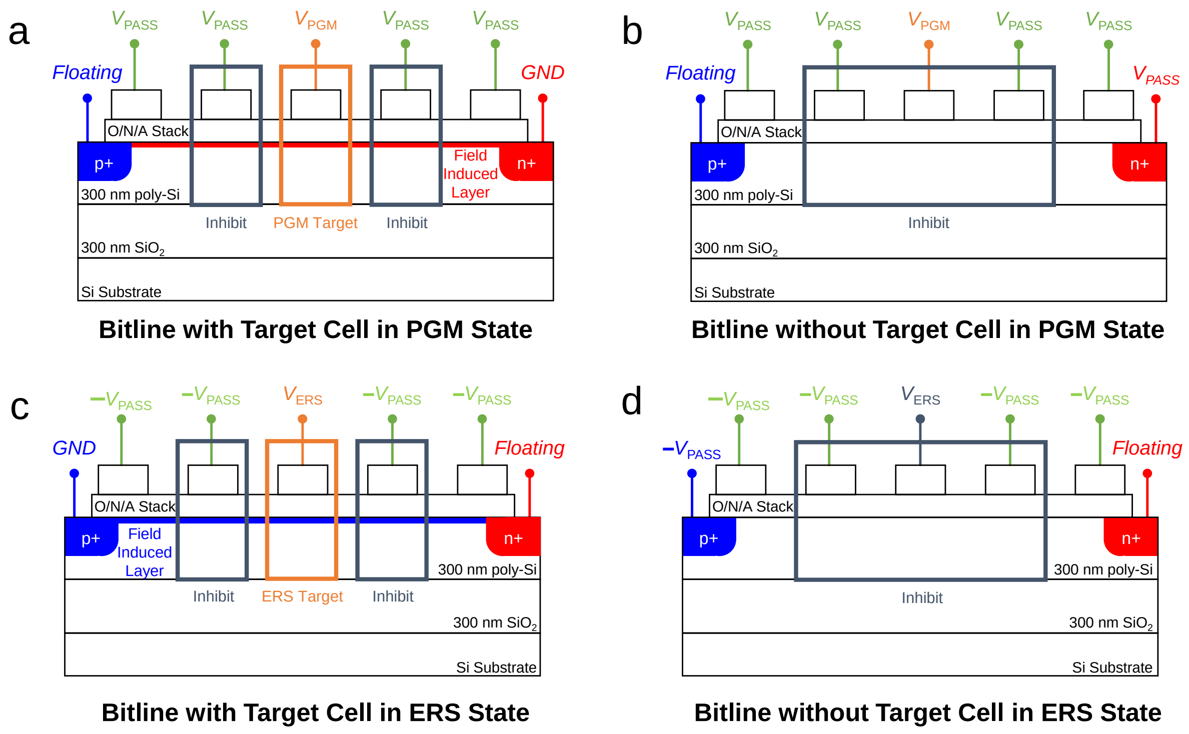

Supplement: Supplementary file 1 — Supporting Information [file ADVS-10-2303817-s001.pdf]
